# Increased levels of inflammatory markers and carotid intima-media thickness in asymptomatic patients with Sheehan syndrome without growth hormone replacement therapy

**DOI:** 10.20945/2359-4292-2024-0140

**Published:** 2025-07-22

**Authors:** Shahnaz Ahmad Mir, Asif Ahmad Naik, Basharat Dar, Hardeep Singh, Bashir Ahmad Laway, Ahila Ashraf, Naseer Ahmad Khan

**Affiliations:** 1 Department of Internal Medicine and Endocrinology, Government Medical College, Srinagar, India; 2 Department of Biochemistry, School of Applied Sciences, Shri Venkateshwara University, Gajraula, Amroha, India; 3 Department of Radiology, Government Medical College, Srinagar, India

**Keywords:** Hypopituitarism, Carotid intima-media thickness, Atherosclerosis

## Abstract

**Objective:**

To evaluate whether inflammatory markers and carotid intima-media thickness are increased in patients with Sheehan syndrome.

**Methods:**

This study included 37 patients diagnosed with Sheehan syndrome who met the eligibility criteria, along with 37 healthy controls matched for age, body mass index, and parity. All participants underwent a detailed clinical evaluation, along with measurement of biochemical and hormonal parameters, as well as inflammatory markers, specifically tumor necrosis factor alpha and interleukin-6. Both patients and controls were assessed for carotid intima-media thickness using a high-resolution color Doppler system.

**Results:**

Patients with Sheehan syndrome had significantly higher mean levels of triglycerides, total cholesterol, and low-density lipoprotein cholesterol, along with lower levels of high-density lipoprotein cholesterol compared with controls. They also exhibited higher levels of tumor necrosis factor alpha (23.41 ± 10.97 pg/mL *versus* 20.05 ± 2.76 pg/mL; p = 0.041) and interleukin-6 (37.19 ± 5.38 pg/mL *versus* 32.08 ± 1.18 pg/mL; p = 0.004), as well as an increased mean carotid intima-media thickness value (0.71 ± 0.07 mm *versus* 0.59 ± 0.05 mm; p = 0.001).

**Conclusion:**

Patients with Sheehan syndrome exhibited risk factors that may elevate their likelihood of developing atherosclerosis.

## INTRODUCTION

Sheehan syndrome is a condition characterized by hypopituitarism, resulting from ischemic necrosis of the pituitary gland due to extreme hypotension or shock caused by severe hemorrhage during or after childbirth (^1^). Although its incidence is believed to have decreased due to improvements in obstetric care, Sheehan syndrome remains one of the most common causes of hypopituitarism in developing countries. The disorder is rare in Western countries, but it continues to be observed in some developing nations (^2^,^3^). An epidemiological survey from the Kashmir valley of the Indian subcontinent has reported a prevalence of Sheehan syndrome of approximately 3% in women older than 20 years, with nearly two-thirds of these women having given birth at home (^4^). Sheehan syndrome is characterized by varying degrees of dysfunction in the anterior and, in some cases, the posterior pituitary gland, manifesting immediately in the postpartum period or after several years, depending on the extent of tissue damage (^5^,^6^).

Patients with hypopituitarism who are receiving appropriate conventional replacement therapy, especially women, have a higher risk of cardiovascular morbidity and mortality compared with the general population (^7^). The exact mechanism behind this increased risk of vascular disease in hypopituitarism remains unclear. However, recent research has called into question the previously held belief that growth hormone (GH) deficiency protects against the risk of cardiovascular events, suggesting instead that GH deficiency may play a role in their pathophysiology (^8^). Notably, GH deficiency, a well-known feature of Sheehan syndrome, is linked to a cluster of cardiovascular risk factors, including abnormalities in lipid and carbohydrate metabolism, insulin resistance, endothelial dysfunction, and increased carotid intima-media thickness (CIMT), among others (^9^-^11^). Moreover, the significant prevalence of coronary calcium deposits in patients with Sheehan syndrome not receiving GH replacement therapy predisposes them to a relatively high risk of coronary heart disease (^12^).

Endothelial dysfunction plays an important role in the pathogenesis of atherosclerosis and can be detected early in individuals at risk for cardiovascular events (^13^). Cytokines, as mediators of inflammation, play a crucial role in endothelial injury (^14^). Several inflammatory markers, including tumor necrosis factor alpha (TNF-α) and interleukin-6 (IL-6), have been shown to predict cardiovascular events in asymptomatic patients who are at high risk for these events. These peripheral markers of inflammatory activity correlate with the level of atherosclerosis detected by CIMT measurement.

Considering the above, the present study aimed to evaluate whether inflammatory markers and CIMT are increased in patients with Sheehan syndrome.

## METHODS

This case-control, cross-sectional observational study was conducted in the Department of Internal Medicine and Endocrinology at Government Medical College Srinagar, following approval from the Institutional Ethics Committee. Written informed consent was obtained from all participants, and the study was conducted in accordance with the principles outlined in the Declaration of Helsinki.

### Study subjects

#### Cases (n = 37)

Women diagnosed with Sheehan syndrome were recruited from the Endocrine Clinic after meeting the study’s inclusion criteria. The diagnosis of Sheehan syndrome was established according to the essential criteria proposed by Diri and cols. (^1^). For this study, cases were defined as women who met the essential criteria for Sheehan syndrome and were stable on conventional replacement treatment without GH replacement for at least 6 months prior to the study. All participants had GH deficiency documented by low levels of insulin-like growth factor 1 (IGF-1) and GH on an insulin tolerance test (ITT), when required.

#### Controls (n = 37)

Healthy women, who were matched by age and body mass index (BMI), from the same ethnic background, and unrelated to those in the case group, served as controls.

#### Inclusion criteria

Women in the postpartum period, regardless of age group, who were hemodynamically stable, had no other comorbidities (e.g., diabetes or hypertension), agreed to participate, and met the above-mentioned criteria for cases and controls, were eligible for the study.

#### Exclusion criteria

The exclusion criteria for all participants included known atherosclerotic disease or established cardiovascular disease, such as hypertension, peripheral arterial insufficiency, a history of myocardial infarction or stroke, and diabetes. Additional exclusions were applied to specific physiological groups, including women who were pregnant or lactating and those at the extremes of age.

#### Method

All study participants underwent anthropometric assessments, which included measurements of weight, height, waist-to-hip circumference, blood pressure, and a detailed physical examination. The participants’ BMI was calculated by dividing body weight (in kg) by the square of height (in m^2^). All patients had been stable on conventional replacement therapy for at least 6 months prior to the study. None of the patients had received GH replacement before. Patients visited the Endocrinology Clinic within the Internal Medicine Department between 8:30 a.m. and 9 a.m., following an overnight fast of 10 to 12 hours and without taking their usual morning replacement therapy. The investigation was conducted on an outpatient basis.

Venous blood samples were collected to measure levels of triglycerides, total cholesterol, high-density lipoprotein (HDL) cholesterol, low-density lipoprotein (LDL) cholesterol, and fasting blood glucose, and to conduct routine hematological tests. Liver and kidney function tests were also conducted. Biochemical analyses, including glucose, total cholesterol, and triglyceride concentrations, were conducted using a Technicon DAX-72 automated analyzer (Technicon, Bayer Corporation, Tarrytown, NY, USA) at the Central Biochemistry Laboratory of the Government Medical College Srinagar. Levels of HDL cholesterol were measured using an automated analyzer (RAXT) following precipitation with phosphotungstic acid and magnesium chloride. Levels of LDL cholesterol were calculated using the Friedwald formula (^15^). Hormonal assays were performed using the electrochemiluminescence method on an Abbott ARCHITECT *i*2000SR immunoassay analyzer, following the manufacturer’s protocol. Levels of IGF-1 were measured using a chemiluminescent immunometric assay (CLIA).

Measurement of CIMT was performed using a high-resolution color Doppler ultrasound system (LOGIQ S8; GE Healthcare, Chalfont St. Giles, UK), following a standard protocol (^16^). Scanning of the extracranial carotid arteries in the neck was conducted bilaterally using three different longitudinal projections, *i.e.*, anterior-oblique, lateral, and posterior-oblique, in addition to a transverse projection. Three CIMT measurements were obtained at the site of greatest thickness, as well as at two additional points: 1 cm proximal and 1 cm distal to that site. The average of these three values was calculated. The highest value among the six averaged CIMT measurements (three from the left side and three from the right side) was used as the representative CIMT value for each individual. The coefficients of variation for the measurements were below 3%. All scans were read by an independent physician who was blinded to the participants’ clinical status.

Serum concentrations of TNF-α and IL-6 were measured in all patients using commercially available enzyme-linked immunosorbent assay (ELISA) kits for cytokine detection (eBioscience ELISA Kits, San Diego, CA, USA), which have an intra-assay coefficient of variation of 5.3 to 8.3%.

#### Statistical analysis

The statistical software Statistical Package for the Social Sciences (SPSS), version 20.0 (SPSS Inc., Chicago, IL, USA), was used to analyze the recorded data. Continuous variables are presented as means ± standard deviations, while categorical variables are summarized as frequencies and percentages. The Shapiro-Wilk test and a normal probability plot were used to assess the normality of the data. Normally distributed continuous variables were compared using Student’s independent *t* test, while non-normally distributed variables were analyzed using the Mann-Whitney U test. A p-value of less than 0.05 was deemed statistically significant. All p-values were calculated using two-tailed tests. Linear regression analysis was conducted to predict the value of one variable based on the value of another variable.

## RESULTS

### Baseline characteristics of the participants

The study included 74 women, 37 with Sheehan syndrome and 37 controls, matched for age, BMI, and parity. Cases and controls were selected to ensure similar distributions in age, BMI, baseline heart rate, systolic blood pressure, and diastolic blood pressure. The details of the biochemical profile and inflammatory markers of the participants are provided in Table 1. Patients received glucocorticoids in the form of divided doses of hydrocortisone, which was the most common treatment (81.08%), or once-daily doses of prednisolone. Combined estrogen-progestin was used as hormone replacement therapy for hypogonadism (67.57%).

**Table 1:** Comparison of anthropometric, clinical, and biochemical characteristics and inflammatory markers among patients with Sheehan syndrome (cases) and controls

Variable	Cases (mean ± SD)	Controls (mean ± SD)	p-value
Age, years	50.1 ± 14.71 (30-80)	47.8 ± 13.54 (29-69)	0.472
Parity	3.4 ± 1.47 (1-7)	2.9 ± 1.28 (1-6)	0.123
HR, beats/minute	73.81 ± 9.76 (51-98)	76.71 ± 14.20 (64-100)	0.231
SBP, mmHg	112.6 ± 8.74 (90-130)	108.9 ± 12.12 (98-126)	0.332
DBP, mmHg	74.6 ± 7.93 (64-84)	70.9 ± 10.34 (70-84)	0.223
BMI, kg/m2	22.08 ± 2.47	21.43 ± 2.04	0.221
Waist-to-hip ratio	0.89 ± 0.10 (0.83-0.99)	0.87 ± 0.09 (0.83-0.98)	0.314
Serum total cholesterol, mg/dL	188.26 ± 45.17	147.53 ± 39.63	< 0.001*
Serum triglycerides, mg/dL	216.29 ± 51.94	154.42 ± 42.17	< 0.001*
Serum LDL cholesterol, mg/dL	112.38 ± 43.82	74.62 ± 28.85	< 0.001*
Serum HDL cholesterol, mg/dL	46.21 ± 10.69	61.35 ± 8.72	<0 .001*
Serum TNF-α, pg/mL	23.41 ± 10.97 (19.8-27.1)	20.05 ± 2.76 (19.1-20.9)	0.041*
Serum IL-6, pg/mL	37.19 ± 5.38 (35.4-38.9)	32.08 ± 1.18 (32.4-33.2)	0.004*
CIMT, mm	0.71 ± 0.07 (0.69-0.74)	0.59 ± 0.05 (0.58-0.61)	< 0.001*

Values in parenthesis are range or 95% confidence interval.

* p-values < 0.05 were considered significant. SD: standard deviation; HR: heart rate; SBP: systolic blood pressure; DBP: diastolic blood pressure; BMI: body mass index; LDL: low-density lipoprotein; HDL: high-density lipoprotein; TNF-α: tumor necrosis factor alpha; IL-6: interleukin-6; CIMT: carotid intima-media thickness.

### Biochemical parameters and inflammatory markers

Patients with Sheehan syndrome, compared with controls, had significantly higher mean levels of triglycerides (216.29 ± 51.94 mg/dL *versus* 154.42 ± 42.17 mg/dL; p < 0.001), total cholesterol (188.26 ± 45.17 mg/dL *versus* 147.53 ± 39.63 mg/dL; p < 0.001), and LDL cholesterol (112.38 ± 43.82 mg/dL *versus* 74.62 ± 28.85 mg/dL; p < 0.001), along with lower levels of HDL cholesterol (46.21 ± 10.69 mg/dL *versus* 61.35 ± 8.72 mg/dL; p < 0.001).

The details of the hormone profiles at the initial evaluation in patients with Sheehan syndrome are presented in Table 2. All patients had at least one hormone deficiency, and 90% of them had multiple hormone deficiencies.

**Table 2:** Baseline hormone levels among patients with Sheehan syndrome

Hormone	Mean ± SD	Normal values	Patients with deficiency (%)
fT3, pg/dL	1.41 ± 0.472	1.7-3.71	45.9
fT4, ng/dL	0.77 ± 0.741	0.59-1.76	51.4
TSH, mIU/L	1.62 ± 2.178	0.5-6.5	51.1
LH, mIU/mL	0.89 ± 1.463	3-12	67.6
FSH, mIU/mL	3.14 ± 1.036	2-6.6	43.2
GH ,ng/mL*	0.17 ± 0.241	>3	100.0
Prolactin, ng/mL*	4.53 ± 2.193	>2	73.0
Cortisol, µg/dL*	3.19 ± 1.356	>20	86.5
IGF-1, ng/mL	18.55 ± 6.12	105-190	100

* Peak values after the insulin tolerance test. Hormone assays were performed with a specific radioimmunoassay. Data are expressed as frequency or median (range or 95% confidence interval), as appropriate, if not mentioned any other way. SD: standard deviation; fT3: free triiodothyronine; fT4: free thyroxine; TSH: thyroid-stimulating hormone; LH: luteinizing hormone; FSH: follicle-stimulating hormone; GH: growth hormone.

Patients with Sheehan syndrome, compared with healthy controls, had significantly higher mean TNF-α levels (23.41 ± 10.97 pg/mL *versus* 20.05 ± 2.76 pg/mL; p = 0.041) and mean IL-6 levels (37.19 ± 5.38 pg/mL *versus* 32.08 ± 1.18 pg/mL; p = 0.004). Additionally, patients with Sheehan syndrome exhibited significantly increased mean CIMT values (0.71 ± 0.07 mm) than controls (0.59 ± 0.05 mm; p < 0.001). Regression analysis showed no significant association between the number of hormone deficiencies and CIMT values (Table 3).

**Table 3:** Results of correlation analysis between carotid intima-media thickness values and the number of hormone deficiencies

Variable	r	p-value
One hormone (8%)	0.251	0.763
Two hormones (21.5%)	0.982	0.016
Three hormones (12%)	0.264	0.816
Four hormones (58.5%)	0.035	0.043

## DISCUSSION

Cardiovascular diseases, the leading cause of death in both developed and developing countries, are not uncommon in patients with hypopituitarism. Reportedly, cardiovascular diseases are considerably more prevalent among patients with hypopituitarism, along with abnormalities in cardiac structure and poor cardiac output (^17^,^18^). Studies have shown that women with hypopituitarism have more than double the risk of cardiovascular mortality compared with the general population (^19^). Patients with Sheehan syndrome experience varying degrees of pituitary insufficiency, which can range from partial to complete hormone insufficiency, with GH deficiency observed in all cases (^5^,^6^). Severe GH deficiency is a well-recognized characteristic of Sheehan syndrome and is associated with a high incidence of cardiovascular morbidity and mortality; this is due to an unfavorable cardiovascular risk profile that includes abnormal body composition, altered lipid profile, reduced quality of life, and osteoporosis (^7^). Replacement with recombinant GH improves most of these altered parameters (^20^). Limited data are available on the effects of GH deficiency in these patients (^21^).

A study has found that patients with Sheehan syndrome who have not previously received GH replacement therapy exhibit a combination of risk factors that contribute to increased cardiovascular risk, ultimately leading to higher morbidity and mortality (^22^). The presence of elevated inflammatory markers, an atherogenic lipid profile, and increased CIMT underscores the importance of preventive measures to reduce cardiovascular risk in patients with Sheehan syndrome. The present study demonstrated that these patients have atherogenic lipid abnormalities characterized by higher levels of triglycerides, total cholesterol, and LDL cholesterol, along with lower levels of HDL cholesterol compared with healthy controls. These observations are consistent with some earlier studies (^23^-^25^). Bülow and cols. reported that adults with untreated GH deficiency had elevated levels of total cholesterol, LDL cholesterol, triglycerides, and apolipoprotein B, and reduced levels of HDL cholesterol compared with healthy adults (^26^). Kelestimur and cols. have reported positive effects of GH replacement on an adverse cardiovascular risk profile, particularly concerning the atherogenic lipid profile, in addition to beneficial effects on quality of life and on lean and fat body mass in patients with Sheehan syndrome (^21^).

In the present study, hormone levels were evaluated in all patients, and each one exhibited at least one hormone deficiency, with 90% presenting multiple hormone deficiencies. Other studies assessing hormone levels following pituitary ischemic infarction have found that 88% of the cases exhibited GH deficiency, 58 to 76% showed gonadotropin deficiency, and 66% demonstrated corticotropin deficiency. In a few earlier studies involving patients with partial Sheehan syndrome, GH deficiency was a universal finding among all women (^5^,^6^). The somatotrophs are located in the lower and lateral regions of the pituitary, which makes them susceptible to damage from ischemic necrosis of the gland; this damage often leads to GH deficiency, a common characteristic observed in the syndrome (^27^,^28^). These hormonal deficiencies, along with inadequate hormone replacement, increase the patients’ metabolic, inflammatory, and cardiovascular risks. Inadequate hormone replacement in these patients increases their therapeutic burden and encourages complacency where none is warranted.

The adult patients with hypopituitarism and untreated GH deficiency in the present study exhibited increased CIMT values, which is a widely recognized, noninvasive indicator of atherosclerosis in the coronary arteries and a predictor of cardiovascular events (^29^-^31^). Studies examining CIMT values in patients with hypopituitarism and untreated GH deficiency have produced conflicting results. While some studies found that these patients had increased CIMT values compared with controls (^32^,^33^), other studies did not confirm this association (^19^,^34^). In the present study, the mean CIMT value was significantly higher in patients with Sheehan syndrome compared with controls matched for age, BMI, and parity. Leonsson and cols. found that patients with GH deficiency had significantly higher CIMT compared with nonobese controls (^35^). Notably, in women, several cardiovascular risk factors were identified that were independent of the degree of adiposity (^35^). The authors noted that a higher waist-to-hip ratio, elevated serum levels of triglycerides, total cholesterol, and LDL cholesterol, along with decreased serum levels of HDL cholesterol in the patients, appeared to be a consequence of GH deficiency. Therefore, the adverse risk profile in the present study may increase the predisposition to adverse cardiovascular events in patients with hypopituitarism due to Sheehan syndrome. The replacement of GH in adult patients with hypopituitarism has been shown to significantly decrease CIMT, suggesting a direct effect of GH on the arterial wall and a potential beneficial impact of GH on the vascular system (^36^). The role of prolactin in cardiovascular risk remains inconclusive. Nevertheless, some studies suggest a connection, as this hormone has been associated with inflammatory processes that play a part in atherosclerosis. In contrast, in a study involving 3,232 participants from the Framingham Heart Study, Therkelsen and cols. found no association between prolactin levels and a comprehensive panel of risk factors for incident cardiovascular disease (^37^).

The results of the present study indicate that patients with Sheehan syndrome have elevated levels of the inflammatory markers IL-6 and TNF-α compared with healthy controls. This increased inflammatory status may contribute to the unfavorable cardiovascular risk profile observed in this population. Potential reasons for the elevated IL-6 and TNF-α levels in women with Sheehan syndrome include both the hormone deficiencies and the effects of hormone replacement therapies. Notably, GH exerts important effects on inflammatory cells, stimulating the production of cytokines. In addition to its direct effects on the production of inflammatory markers, GH may also have important indirect effects by altering body composition. Increased production of IL-6 and TNF-α from monocytes, along with increased concentrations of these cytokines in the peripheral serum, have been observed in patients with GH deficiency. Furthermore, GH replacement has been associated with a decrease in the levels of these cytokines, suggesting that GH may play a role in regulating inflammation in the vascular wall. We propose that GH deficiency could be a contributing factor involved in regulating cytokine production in patients with Sheehan syndrome, as demonstrated in our study. The increased adipose tissue mass in patients with GH deficiency may also contribute to the synthesis of TNF-α and lead to elevated levels of inflammatory markers in these patients. Our study emphasizes the importance of further investigating the role of GH deficiency and its treatment in patients with Sheehan syndrome.

The present study showed that patients with Sheehan syndrome who are receiving conventional hormone replacement without GH exhibit a clustering of adverse cardiovascular risk factors, *i.e.*, an atherogenic lipid profile, elevated levels of the inflammatory markers TNF-α and IL-6, and increased CIMT values compared with healthy controls. In our region, Sheehan syndrome is one of the most common causes of adult GH deficiency and may be linked to an increased cardiovascular risk profile in these patients. Further research is needed to explore the underlying pathophysiological mechanisms and implications of this association.

This paper was presented at the 32nd Annual American College of Clinical Endocrinology Meeting in San Diego, USA, in 2022, as an oral presentation, with the abstract listed as #1184265.

## References

[1] Diri H, Karaca Z, Tanriverdi F, Unluhizarci K, Kelestimur F (2016). Sheehan’s syndrome: new insights into an old
disease.. Endocrine..

[2] Kilicli F, Dokmetas HS, Acibucu F (2013). Sheehan’s syndrome. Gynecol Endocrinol Off J Int Soc Gynecol Endocrinol..

[3] Keleştimur F (2003). Sheehan’s syndrome. Pituitary.

[4] Zargar AH, Singh B, Laway BA, Masoodi SR, Wani AI, Bashir MI (2005). Epidemiologic aspects of postpartum pituitary hypofunction
(Sheehan’s syndrome). Fertil Steril..

[5] Laway BA, Mir SA, Gojwari T, Shah TR, Zargar AH (2011). Selective preservation of anterior pituitary functions in
patients with Sheehan’s syndrome. Indian J Endocrinol Metab.

[6] Laway B, Misgar R, Mir S, Wani A (2016). Clinical, hormonal and radiological features of partial Sheehan’s
syndrome: an Indian experience. Arch Endocrinol Metab..

[7] Bülow B, Hagmar L, Eskilsson J, Erfurth EM (2000). Hypopituitary females have a high incidence of cardiovascular
morbidity and an increased prevalence of cardiovascular risk
factors. J Clin Endocrinol Metab..

[8] de Gennes JL, Turpin G, Heshmati HM, Lebrun A (1979). [Hypopituitarism and hyperlipidemia. Protective effect of growth
hormone deficiency against atherosclerosis (author’s
transl)]. Ann Endocrinol..

[9] Merimee TJ, Hollander W, Fineberg SE (1972). Studies of hyperlipidemia in the HGH-deficient
state. Metabolism..

[10] Rosén T, Edén S, Larson G, Wilhelmsen L, Bengtsson BA (1993). Cardiovascular risk factors in adult patients with growth hormone
deficiency. Acta Endocrinol (Copenh)..

[11] Hew FL, Koschmann M, Christopher M, Rantzau C, Vaag A, Ward G (1996). Insulin resistance in growth hormone-deficient adults: defects in
glucose utilization and glycogen synthase activity. J Clin Endocrinol Metab..

[12] Singh H, Afroze M, Shafi N, Bhat JA, Kawa IA, Laway BA (2021). Prevalence of coronary calcium deposits in Sheehan’s syndrome
patients on long term replacement treatment. Pituitary..

[13] Lüscher TF, Barton M (1997). Biology of the endothelium. Clin Cardiol..

[14] Nawroth PP, Stern DM (1986). Modulation of endothelial cell hemostatic properties by tumor
necrosis factor. J Exp Med..

[15] Knopfholz J, Disserol CC, Pierin AJ, Schirr FL, Streisky L, Takito LL (2014). Validation of the friedewald formula in patients with metabolic
syndrome. Cholesterol..

[16] Terminology and Diagnostic Criteria Committee, Japan Society of
Ultrasonics in Medicine (2009). Standard method for ultrasound evaluation of carotid artery
lesions. J Med Ultrason (2001)..

[17] Abdu TA, Elhadd T, Pfeifer M, Clayton RN (2001). Endothelial dysfunction in endocrine disease. Trends Endocrinol Metab TEM..

[18] Laway BA, Ramzan M, Allai MS, Wani AI, Misgar RA (2016). Cardiac structural and functional abnormalities in females with
untreated hypopituitarism due to sheehan syndrome: response to hormone
replacement therapy. Endocr Pract..

[19] Bülow B, Hagmar L, Eskilsson J, Erfurth EM (2000). Hypopituitary females have a high incidence of cardiovascular
morbidity and an increased prevalence of cardiovascular risk
factors. J Clin Endocrinol Metab..

[20] Maison P, Griffin S, Nicoue-Beglah M, Haddad N, Balkau B, Chanson P (2004). Impact of growth hormone (GH) treatment on cardiovascular risk
factors in GH-deficient adults: a Metaanalysis of Blinded, Randomized,
Placebo-Controlled Trials. J Clin Endocrinol Metab..

[21] Kelestimur F, Jonsson P, Molvalilar S, Gomez JM, Auernhammer CJ, Colak R (2005). Sheehan’s syndrome: baseline characteristics and effect of 2
years of growth hormone replacement therapy in 91 patients in KIMS - Pfizer
International Metabolic Database. Eur J Endocrinol..

[22] Bülow B, Hagmar L, Eskilsson J, Erfurth EM (2000). Hypopituitary females have a high incidence of cardiovascular
morbidity and an increased prevalence of cardiovascular risk
factors. J Clin Endocrinol Metab..

[23] Mir SA, Shah T, Singh H, Shabir I, Laway BA (2018). Serum Lipid and Leptin Concentrations in Patients with Sheehan
Syndrome. Indian J Endocrinol Metab..

[24] Bhat MA, Laway BA, Shah ZA, Wani AI, Mubarik I (2015). Insulin resistance, metabolic syndrome and chronic low grade
inflammation in Sheehan’s syndrome on standard replacement therapy: a case
control study. Pituitary..

[25] Ozbey N, Algun E, Turgut AS, Orhan Y, Sencer E, Molvalilar S (2000). Serum lipid and leptin concentrations in hypopituitary patients
with growth hormone deficiency. Int J Obes Relat Metab Disord J Int Assoc Study Obes..

[26] Bülow B, Hagmar L, Mikoczy Z, Nordström CH, Erfurth EM (1997). Increased cerebrovascular mortality in patients with
hypopituitarism. Clin Endocrinol (Oxf)..

[27] Zargar AH, Masoodi SR, Laway BA, Shah NA, Salahuddin M, Siddiqi MA (1996). Clinical spectrum of Sheehan’s syndrome. Ann Saudi Med..

[28] Dökmetaş HS, Kilicli F, Korkmaz S, Yonem O (2006). Characteristic features of 20 patients with Sheehan’s
syndrome. Gynecol Endocrinol..

[29] Wofford JL, Kahl FR, Howard GR, McKinney WM, Toole JF, Crouse JR (1991). Relation of extent of extracranial carotid artery atherosclerosis
as measured by B-mode ultrasound to the extent of coronary
atherosclerosis. Arterioscler Thromb J Vasc Biol..

[30] Hulthe J, Wikstrand J, Emanuelsson H, Wiklund O, de Feyter PJ, Wendelhag I (1997). Atherosclerotic changes in the carotid artery bulb as measured by
B-mode ultrasound are associated with the extent of coronary
atherosclerosis. Stroke..

[31] O’Leary DH, Polak JF, Kronmal RA, Manolio TA, Burke GL, Wolfson SK (1999). Carotid-artery intima and media thickness as a risk factor for
myocardial infarction and stroke in older adults. Cardiovascular Health
Study Collaborative Research Group. N Engl J Med..

[32] Pfeifer M, Verhovec R, Zizek B, Prezelj J, Poredos P, Clayton RN (1999). Growth hormone (GH) treatment reverses early atherosclerotic
changes in GH-deficient adults. J Clin Endocrinol Metab..

[33] Markussis V, Beshyah SA, Johnston DG, Fisher C, Nicolaides AN, Sharp P (1992). Detection of premature atherosclerosis by high-resolution
ultrasonography in symptom-free hypopituitary adults. Lancet..

[34] Elhadd TA, Abdu TA, Oxtoby J, Kennedy G, McLaren M, Neary R (2001). Biochemical and biophysical markers of endothelial dysfunction in
adults with hypopituitarism and severe GH deficiency. J Clin Endocrinol Metab..

[35] Leonsson M, Hulthe J, Oscarsson J, Johannsson G, Wendelhag I, Wikstrand J (2002). Intima-media thickness in cardiovascularly asymptomatic
hypopituitary adults with growth hormone deficiency: relation to body mass
index, gender, and other cardiovascular risk factors. Clin Endocrinol (Oxf)..

[36] Borson-Chazot F, Serusclat A, Kalfallah Y, Ducottet X, Sassolas G, Bernard S (1999). Decrease in carotid intima-media thickness after one year growth
hormone (GH) treatment in adults with GH deficiency. J Clin Endocrinol Metab..

[37] Therkelsen KE, Abraham TM, Pedley A, Massaro JM, Sutherland P, Hoffmann U, Fox CS (2016). Association Between Prolactin and Incidence of Cardiovascular
Risk Factors in the Framingham Heart Study. J Am Heart Assoc..

